# Options for modifying UK alcohol and tobacco tax: A rapid scoping review of the evidence over the period 1997–2018

**DOI:** 10.3310/nihropenres.13379.3

**Published:** 2023-10-30

**Authors:** Jenny Hatchard, Penny Buykx, Alan Brennan, Duncan Gillespie

**Affiliations:** 1 Population Health Sciences, Bristol Medical School, University of Bristol, Bristol, UK; 2 Tobacco Control Research Group, Department for Health, University of Bath, Bath, UK; 3 School of Humanities and Social Science, University of Newcastle, Australia, New South Wales, Australia; 4 Sheffield Centre for Health and Related Research (SCHARR), Division of Population Health, School of Medicine and Population Health, University of Sheffield, Sheffield, UK

**Keywords:** commercial influences on health, unhealthy commodities, public health

## Abstract

**Background:**

Increased taxation is recognised worldwide as one of the most effective interventions for decreasing tobacco and harmful alcohol use, with many variations of policy options available. This rapid scoping review was part of a NIHR-funded project (‘SYNTAX’ 16/105/26) and was undertaken during 2018 to inform interviews to be conducted with UK public health stakeholders with expertise in alcohol and tobacco pricing policy.

**Methods:**

Objectives: To synthesise evidence and debates on current and potential alcohol and tobacco taxation options for the UK, and report on the underlying objectives, evidence of effects and mediating factors. Eligibility criteria: Peer-reviewed and grey literature; published 1997–2018; English language; UK-focused; include taxation interventions for alcohol, tobacco, or both. Sources of evidence: PubMed, Scopus, Cochrane Library, Google, stakeholder and colleague recommendations.

**Charting methods:**

Excel spreadsheet structured using PICO framework, recording source characteristics and content.

**Results:**

Ninety-one sources qualified for inclusion: 49 alcohol, 36 tobacco, 6 both. Analysis identified four policy themes: changes to excise duty within existing tax structures, structural reforms, industry measures, and hypothecation of tax revenue for public benefits. For alcohol, policy options focused on raising the price of cheap, high-strength alcohol. For tobacco, policy options focused on raising the price of all tobacco products, especially the cheapest products, which are hand-rolling tobacco. For alcohol and tobacco, there were options such as levies that take money from the industries to help reduce the societal costs of their products. Due to the perceived social and economic importance of alcohol in contrast to tobacco, policy options also discussed supporting pubs and small breweries.

**Conclusions:**

This review has identified a set of tax policy options for tobacco and alcohol, their objectives, evidence of effects and related mediating factors. The differences between alcohol and tobacco tax policy options and debates suggest an opportunity for cross-substance policy learning.

## Introduction

Alcohol and tobacco are major risk factors for a wide range of diseases ^1, 2^. To give a sense of the scale of the health burden caused by alcohol and tobacco consumption, in 2016 in England, there were 337,000 alcohol-related hospital admissions, and 484,700 tobacco-related hospital admissions ^3, 4^. Furthermore, drinking and smoking are correlated behaviours ^5^, meaning that people who smoke tend to have higher levels of alcohol consumption, and these people consequently spend a high proportion of their budgets on alcohol and tobacco ^6^. Of the available interventions to jointly address alcohol and tobacco related harms ^7^, increasing taxes on alcohol and tobacco is considered among the most effective approaches to improve health ^8, 9^. In general, there is evidence that increases in product prices decrease demand ^10– 16^, especially in socio-economically disadvantaged groups ^17– 19^.

Excise taxes are those levied on selected goods produced for sale in a country or imported and sold in that country. They are imposed by governments mainly as specific excise taxes or ad valorem taxes, and are collected from the producer or manufacturer within a certain time frame (e.g., 20–30 days) after the product has left the factory ^20^. Excise duties are commonly applied to alcohol (155 countries in 2016) and tobacco products (166 countries in 2018) worldwide ^21, 22^. As elsewhere, excise duties are applied in the UK after manufacturing. Both alcohol and tobacco products incur specific excise duties (calculated as a fixed tax per specified element of product: e.g., per litre, cigarette stick, gram). Cigarettes also incur ad valorem excise duties (calculated as a percentage of the retail price) and, since 2017, have been subject to a minimum excise duty (set at £293.95 per 1,000 cigarettes at the time that this review was conducted, but having subsequently increased). Since 2010, tobacco excise duty has been increased annually in accordance with a duty escalator of a certain percentage above inflation (the rate of increase in tobacco duty under the escalator has varied over time). The UK Government decided to remove a similar duty escalator for alcohol in 2013 (for beer) and 2014 (for other alcohol products). The report by Angus and Henney ^23^ gives model-based estimates of the impact of the decision to remove the alcohol duty escalator and of the subsequent reductions and freezes in alcohol duty. The rates of excise duties in the UK at the time that this review was conducted can be found in Box 1 ^24, 25^. At that time, UK alcohol and tobacco tax options were constrained by shared European Union (EU) directives on alcohol and tobacco tax ^26, 27^, which have since been passed across into UK law. In addition to excise duties, alcohol and tobacco products in the UK are also subject to a 20% tax applied at point of sale (known in the UK as Value Added Tax (VAT)).

**Table:** Box 1. UK excise duty rates and European Union duty regulations for alcohol and tobacco (correct for January 2019)

	UK Excise duty rates as at January 2019	European Union regulations
TOBACCO		
Cigarettes	The highest of: 16.5% of the retail price plus £4.57 on a packet of 20 OR £293.95 per 1,000 cigarettes (Minimum Excise Tax)	Directive 2011/64/EU requires Member States to levy a minimum rate of excise duties on cigarettes which must consist of: - A specific component of between 7.5% and 76.5% of the total tax burden (TTB) - expressed as a fixed amount per 1000 cigarettes - An ad valorem component - expressed as a percentage of the maximum retail selling price In addition, the overall excise rate must be: - At least EUR 90 per 1000 cigarettes - At least 60% of the weighted average retail selling price [Only applies to Member States that apply excise duty < EUR 115].
Cigars	£2.85 on a 10g cigar	Directive 2011/64/EU requires Member States to levy a minimum rate of excise duties on other tobacco products. Member States can choose between applying a specific component or an ad valorem component, or if they wish, they may apply a mixture of the two. - Fine-cut smoking tobacco: 48% (rising to 50% by 2020) of the weighted average retail selling price OR EUR 60 per kilogram* - Cigars and Cigarillos: 5% of the retail selling price OR EUR 12 per 1000 or per kilogram - Other smoking tobaccos: 20% of the retail selling price OR EUR 22 per kilogram
Hand rolling tobacco	£5.87 on a 25g packet	
Other smoking tobacco and chewing tobacco	£3.13 on a 25g packet	
ALCOHOL		
Beer >1.2% - ≤2.8%	8.42p per litre for each % alcohol	Hectolitre per degree Plato: EUR 0.748 OR Hectolitre per degree alcohol: EUR 1.87
Beer >2.8% - ≤7.5%	19.08p per litre for each % alcohol	
Beer >7.5%	24.77p per litre for each % of alcohol	
Still cider >1.2% - ≤7.5%	40.38p per litre	Standard VAT rate, which cannot be less than 15%.
Still cider >7.5% - <8.5%	61.04p per litre	
Sparkling cider >1.2% - ≤5.5%	40.38p per litre	
Sparkling cider >5.5% - <8.5%	279.46p per litre	
Still wine >1.2% - ≤4%	88.93p per litre	Wine (still or sparkling) Hectolitre of volume: EUR 0 Intermediate products (e.g. sherry or port) Hectolitre of volume: EUR 45
Still wine >4% - ≤5.5%	122.30p per litre	
Still wine >5.5% - ≤15%	288.65p per litre	
Still wine >15% - ≤22%	384.82p per litre	
Sparkling wine >5.5% - <8.5%	279.46p per litre	
Sparkling wine >8.5% - ≤15%	369.72p per litre	
Spirits	2874p per litre of pure alcohol	Hectolitre of pure alcohol: EUR 550

[sources: UK: https://www.gov.uk/government/publications/rates-and-allowances-excise-duty-tobacco-duty/excise-duty-tobacco-duty-rates; https://www.gov.uk/government/publications/rates-and-allowance-excise-duty-alcohol-duty/alcohol-duty-rates-from-24-march-2014, EU: https://ec.europa.eu/taxation_customs/business/excise-duties-alcohol-tobacco-energy/excise-duties-alcohol_en; https://ec.europa.eu/taxation_customs/business/excise-duties-alcohol-tobacco-energy/excise-duties-tobacco_en

Since conducting this review, the UK government has decided to introduce changes to the duty structure for alcohol products, creating a standardised series of tax bands based on alcohol by volume and introducing tax relief for small producers and on products sold in the on-trade venues, such as pubs ^28^. These reforms came into effect on 1 ^st^ August 2023.

The objective of this scoping review was to synthesise evidence and debates on contemporary alcohol and tobacco taxation options for the UK, and to report underlying objectives, observed or predicted effects and mediating factors. It was undertaken to inform subsequent interviews conducted with UK public health stakeholders with expertise in alcohol and tobacco pricing policy. These interviews were undertaken in 2018 as part of the progression of research in the National Institute for Health and Care Research funded ‘SYNTAX’ project (16/105/26) ^29^. The SYNTAX project aimed to produce the evidence required for joint policy analysis of alcohol and tobacco tax policy changes. Therefore, both existing policies and ideas for policy innovation were in scope for this review.

## Methods

### Patient and public involvement

Patients and the public were not involved in any way for this study.

### Ethics

Whilst ethical approval was not required specifically for this scoping review of the literature, this review formed part of work package 1 of the SYNTAX project, which conducted qualitative research on alcohol and tobacco tax policy interventions. Ethical approval for the qualitative research element of work package 1 was obtained from the Sheffield University, UK, Sheffield Centre for Health and Related Research Ethics Committee (ref. 017409, 2018) and confirmed by the REACH Committee at the University of Bath, UK.

### Review approach

We undertook a scoping review of alcohol and tobacco tax policy research in the UK (see Arksey and O’Malley ^30^ for a discussion of the variation in scoping review methods). We followed a modified version of the rapid review approach recommended by Tricco *et al.*, ^31, 32^. This involved searching >1 database, published and grey literature, searches limited by date and language, research scope specified by two researchers in consultation with the SYNTAX project team and a health librarian, study selection by one reviewer only, data abstraction by one reviewer and one verifier. Modifications to suit the scoping nature of our study were inclusion of grey literature (enabling us to gather wider information about tax options) and exclusion of quality appraisal, which was extraneous to the study’s research objectives: namely, to identify and describe policy options for tobacco and/or alcohol tax. Due to the scoping nature of our study, we simply distinguish studies that are qualitative from studies that present policy effect sizes, which could be effect sizes observed from statistical analyses or effect sizes predicted by computer simulation models. Whilst we acknowledge that observed and predicted effect sizes are not equivalent forms of evidence, it was outside the scope of this study to quality appraise and synthesise the effect size evidence. This method was chosen because it simplifies the systematic review process to produce a synthesis of available knowledge more quickly ensuring feasibility and timeliness, while minimising risk of bias ^31, 33^. It was also chosen as a way to produce a briefing for policy stakeholders and support policy discussion ^34^. For the study’s full protocol, see *Extended data* ^35^.

### Eligibility criteria

Inclusion was restricted to peer-reviewed and grey literature, including governmental, non-governmental and alcohol and tobacco industry documents, published 1997–2018, written in English. Sources had to include a specific description and/or analysis of one or more historical, current, or prospective taxation interventions for either alcohol, tobacco, or both for the UK. Documents which had a global focus but referred briefly to the UK or referred only to non-specific interventions, such as “increase tax”, were excluded.

### Information sources

Searches were conducted in April 2018 using the PubMed, Scopus and Cochrane Library databases, supplemented by a Google grey literature search. Additional sources proposed by stakeholders and colleagues were located and screened against the inclusion criteria in December 2018.

### Search process

Search terms used were ((tobacco OR cig* OR alcohol OR beer OR wine OR cider OR spirits) AND (tax OR taxes OR taxation OR excise OR duty) AND (UK OR “United Kingdom” OR Scotland OR England OR Wales OR “Northern Ireland” OR Britain)). Google searches also included (AND pdf) to help restrict the volume of documents arising from the search to reports, rather than webpages. The search process and terms used were discussed with a subject librarian at the University of Bath and agreed by the project team. A summary of searches undertaken and associated results can be found in *Extended data* ^35^. Search results from databases were extracted directly to the Endnote reference management software (free alternatives such as Mendeley are available). Documents from Google searches, stakeholders and colleagues were recorded in a spreadsheet and transferred manually.

### Selection of sources of evidence

Duplicates were removed automatically using reference management software. Titles and abstracts for the remaining results were screened against the inclusion criteria. Full texts of the remaining documents were screened.

### Data charting

An excel spreadsheet structured according to the PICO framework was used to extract data. The PICO framework is a commonly used model for structuring the key elements of studies in terms of the Population, Intervention, Comparator, Outcome. JH and PB designed and tested the spreadsheet. Data were charted by JH. Charting was monitored via regular peer debriefing with PB and DG and minor modifications were made. For example, additional columns were added to record which categories of tax policy options were referred to in sources as those categories emerged from the data.

### Data items

Reference information (reference number, title, author, date), type of source, declared funding, aims, methods and findings were charted where information was available. The PICO framework was then used to chart data content as follows. For Population, we charted geographical context, population group and timeframe in which the intervention was posited or implemented, product type and sub-type. For Intervention, we charted the technical description of the tax intervention and objectives. For Comparator, we charted the system of taxation, or aspect of the system, that was or would be changed by the intervention. Finally, for Outcome, we charted primary effects (e.g., product price or consumer behaviour); secondary effects (e.g., on health, social and economic outcomes), differential effects among subgroups, and mediating factors which could impact effectiveness.

### Synthesis of results

Extracted data were synthesised across the data set. Similar taxation interventions were grouped together and described in terms of their objectives, observed or predicted effects and mediating factors. JH used an inductive approach to identify categories within each of these components, and categories were refined via discussion with PB and DG. In each subsection, we report the proportion of sources that were peer-reviewed journal articles. Due to the inclusion of published and grey literature, sources varied widely in the types of evidence they presented or cited. We sought to reflect this variation in the wording of our results by, for example, indicating where cited examples were based on evaluative or predictive studies and by using associated terms such as ‘observed’ or ‘predicted’.

## Results

### Selection of sources of evidence

**Figure 1.**
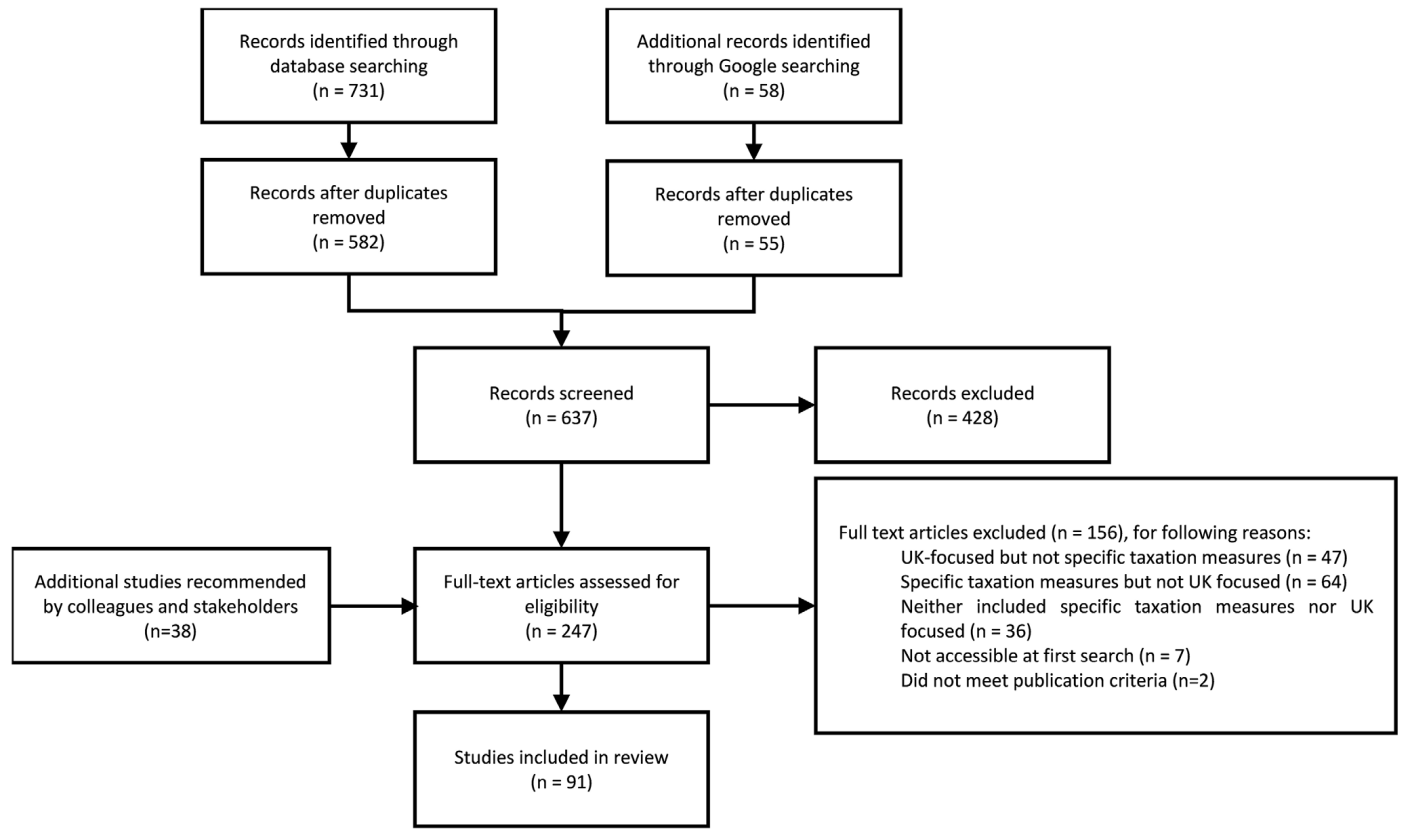
Source search record.

Figure 1 shows the source search record. Database searches returned 731 documents; the Google search returned 58 documents; a further 38 documents were identified through conversations with the project team and stakeholders. After duplicates were removed, 637 documents were screened at the title and abstract stage; and 247 full texts were screened. Of these, 91 sources met the inclusion criteria.

### Characteristics and coverage of sources of evidence

This study’s *Extended data* ^35^ provides detailed characteristics of all sources included in the study. Included sources were published between 1997 and 2018 and comprised 33 peer-reviewed journal articles and 58 grey literature sources. Methods were described by authors in just over half of sources and were predominantly quantitative in nature (e.g., predictive modelling studies, trends analyses, descriptive analyses). Funding was declared in 62 sources: nearly half of which were funded by government or research councils and only 4 by industry. Alcohol was the focus of 49 sources, 36 were on tobacco, 6 on both alcohol and tobacco.

### Policy options identified

Policy options identified were grouped thematically into four categories:

1. *Changes to excise duty* to increase product prices using existing tax structures.
2. *Structural reforms* to the tax applied to alcohol and tobacco products.
3. *Industry measures* – tax changes designed to modify the revenue or profits that the alcohol and tobacco industries gain from product sales.
4. *Hypothecation motives for changing tax* (applied to options 1 to 3 above) – increasing tax in order to spend the revenue on public benefits, especially on initiatives that further the reduction of harmful alcohol and tobacco consumption or mitigate its harmful effects.

**Figure 2.**
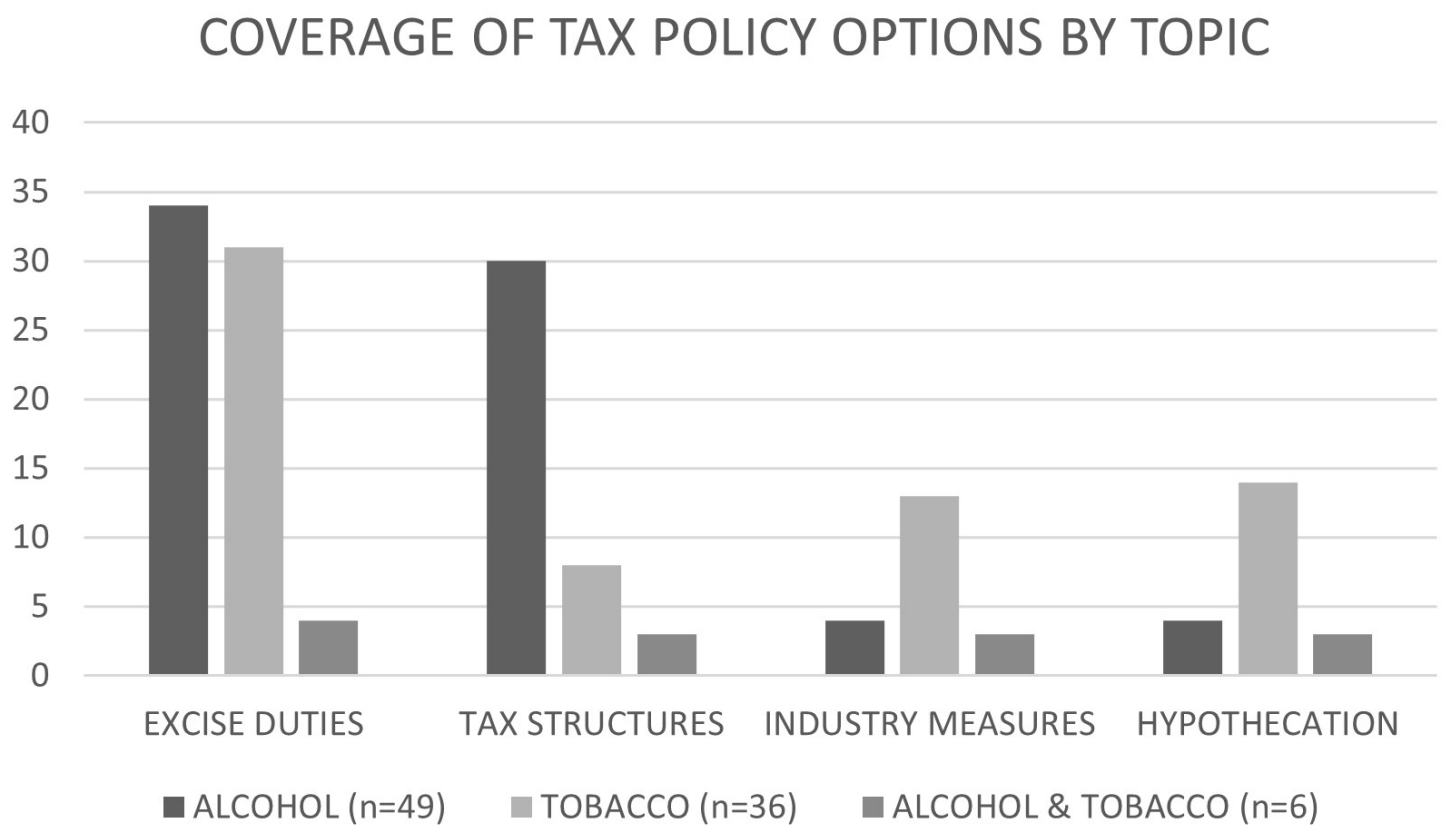
Coverage of tax policy options in sources, n=91.

Figure 2 and Table 1 show a breakdown of the sources that refer to each of the policy options. In Table 1, the sources indicated in bold presented policy effect sizes, which could be observed from statistical analyses of real-world policy effects or predicted by computer simulation models of policy effects. Observed or predicted effects of the policy options described in the included literature were reported in 48 sources, 23 of which were peer-reviewed journal articles. The nature and evidence of effects are summarised below.

**Table 1.** Overview of studies by tax policy option. Bold indicates studies that presented policy effect sizes.

Category	Sub-category	No. studies	Alcohol (n=49)	Tobacco (n=36)	Alcohol/ Tobacco (n=6)
Excise duty	Excise duty rates	37	**19**, **36**, **37**, **38**, **46**, **47**, 48, **49**, **50**, 51, 52, 53, 54, 55, 56, 57, 58, 59, 60	**39**, **40**, **41**, **42**, **43**, **44**, **45**, **61**, **62**, **63**, 64, 65, 66, 67, 68, **69**	**70**, 71
	Duty escalators	33	17, **46**, **47**, **48**, 55, 60, **72**, **73**, **74**, **75**, **76**, **77**, **78**, 79, 80, 81, 82	39, **61**, 65, 67, **83**, **84**, **85**, 86, 87, 88, 89, 90, 91, **92**, 93	94
	Product-specific excise duty	24	**19**, 46, 51, 53, 55, 58, 74, 78, 79, 80, 81, 95, 96, 97	**64**, 65, **83**, 86, 87, 88, 90, **98**, 99	100
	Minimum excise duty	9	No data	64, 65, 83, 86, 87, 88, 90, 101, 102	No data
Taxation structures	Equivalent taxation (per unit,per gram)	22	**36**, 38, 46, 48, 56, 81, 82, 95, **103**, 104, 105, 106, 107, 108, 109, 110, 111	64, 90, 101, 102	**70**
	Multi-rate taxation (rates set according to product strength or harm)	22	**72**, 74, 75, **76**, 46, 52, 54, 55, 81, **95**, **103**, **104**, **105**, 106, 111, 112, 113, 114	86, 89, 115	94
	Supplementary tax (in addition to excise duty & VAT)	4	**36**, 54, **116**	No data	**117**
	International harmonisation (reduce cross-border price gaps)	4	49, 96	40, 86	No data
Industry measures	Industry levy (for retailers or manufacturers)	12	No data	65, 86, 87, 88, 89, **118**, 119, 120, **121**	70, **117, 122**
	Wholesale price cap (limiting profitability of manufacturers)	6	No data	90, 91, 101, 102, 118, 121	No data
	Industry subsidy (to support particular producers or retail sectors)	3	72, **123, 124**	No data	No data
Hypothecation	Hypothecation for prevention or treatment services or the NHS	21	38, 52, 53, **116**	**45**, 61, 62, 65, 66, 67, **83**, 86, 87, 88, 92, 118, 119, 120	70, **117**, 122

### Changes to excise duty

***Changes to excise duty rates*.** Thirty-seven sources: Potential changes discussed in the literature ranged from −2% to 34% for alcohol and 2% to 25% for tobacco. Estimates of policy effects were presented in 20 out of 37 sources: 8 alcohol, 11 tobacco and 1 cross-sector (shown in bold in Table 1).

For alcohol, examples in the literature of modelled effects of tax-led price change on population-level consumption include: a 10% price increase leading to a 4.4% reduction in consumption ^19^; a 13.4% increase in all alcohol duty leading to a 1.7% reduction in consumption ^36^. These two studies also examined estimated impacts on particular groups in society and showed consumption was predicted to fall most for ‘hazardous’ and ‘harmful’ drinkers, with the latter likely to incur greatest additional expenditure from the policies. The second study also estimated a shallow socio-economic gradient with those in the lowest quintile predicted to reduce consumption by 2.3% versus 1.1% for the highest quintile. Modelling also showed that tax-related price increases of 1% above inflation are likely to reduce violence-related hospital emergency department visits ^37, 38^.

For tobacco, there is observed evidence of reduced cigarette sales ^39^ and consumption ^40, 41^ arising from excise duty increases. These effects occur by encouraging quitting and switching behaviours ^42, 43^, and can have a greater effect on younger consumers ^44^. For example, a 1% increase in price was reported to reduce consumption by 0.5% ^41^ and modelling has shown that between 1998 and 2010, an estimated 31% of falling tobacco product consumption can be attributed to increased price ^44^. As with alcohol, increased tobacco duty rates may also impact healthcare costs. A modelled tobacco duty increase of 10% in Scotland was predicted to save an estimated £17m in prevented hospitalisations over ten years if the revenue raised was subsequently hypothecated for tobacco control measures targeted at the most deprived quintile ^45^.

***Duty escalators*.** Thirty-three sources: Policy options covered in the literature were duty escalators that increase alcohol and tobacco duty annually by between 2% and 5% above inflation. Estimates of the effects of escalators were presented in 15 out of 33 sources: 10 alcohol and 5 tobacco.

For alcohol, a 2% duty escalator was in place in the UK between 2008 and 2013, enabling effects to be observed rather than modelled. The escalator reduced affordability of alcohol products by between 22% (beer) and 54% (wine) ^72, 73^, a trend which reversed after the policy ended ^72, 74^. The escalator’s abolition in 2013/14 was observed to decrease alcohol duty in real terms ^75– 77^, reduce prices ^46, 78^ and increase affordability (particularly of cheap strong drinks and off-trade purchases – e.g. beer was 21.8% more affordable, spirits were 14.2% more affordable) ^47, 74, 76, 77^. It also significantly reduced government revenue ^70, 72, 74– 76^. Despite this evidence, the Scotch Whisky Association predicted that scrapping the duty escalator would create jobs and generate public revenue ^48^.

For tobacco, the duty escalator has been observed to reduce smoking rates among lower income smokers ^61^. Modelled analysis of a 5% above inflation tobacco duty escalator from 2015 estimated that by 2035 prices would be 87.6% (for factory-made cigarettes (FM)) and 78.2% (for hand-rolled tobacco (HRT)) higher than under the existing 2% escalator ^83^. The relative effects of duty increases and escalators were compared in 13 sources. Hospital admissions and mortality rates have been shown to be affected by both alcohol and tobacco duty rates and escalators ^45– 47, 49, 62, 72, 73, 84^. These policy options also generate revenue ^61, 63, 84, 85^ and long-term savings to society ^45, 47, 50, 83^.

***Change duty rates on specific products*.** Twenty-four sources: Policy options for alcohol included raising excise rates on stronger and more harmful ciders, beers, spirits and wines, and alcohol products retailing at less than £0.30 per unit. For tobacco, measures included raising excise duty rates 2% above inflation for FM and 5–10% above inflation for HRT and pipe tobacco in order to close the price gap with FM. Estimates of effects were presented in 4 out of 24 sources: 1 alcohol and 3 tobacco.

For alcohol, one modelling study showed the effect of using tax to increase the price of products retailing at <£0.30 per unit by 25% ^19^. For on-trade products, the model predicted a fall of 1.3% in overall consumption, with hazardous drinkers most affected and a small increase in spending of £3.10–£24.00 per annum per drinker. For off-trade products, the model predicted a fall of 0.6% in overall consumption, with hazardous drinkers most affected and a small increase in spending of £2.10–£37.80 per annum per drinker.

For tobacco, evidence relates to HRT: a 2011 duty increase on HRT increased prices, narrowing the price gap with FM ^64^. This is important because increases in the UK’s tax gap between FM and HRT are associated with an increase in the proportion and number of smokers who smoke HRT ^98^ and increased product price is expected to reduce smoking uptake ^83^.

***Minimum excise tax (MET)*.** Nine sources: MET was introduced in the UK in 2017 for FM. It sets a minimum level of excise duty based on the weighted average price of tobacco. The intention of the policy was that the minimum level of excise duty is uprated annually at every budget. At the time of our search there were no papers examining the actual effects of MET. However, 6 papers raised the possibility of introducing a minimum consumption tax. This would extend MET to include value added tax (VAT) and would thereby impact on the price of HRT ^64, 65, 84, 86– 88^.

### Structural reforms

***Equivalent taxation*.** Twenty-two sources: Equivalent taxation is where a universal rate of duty is applied per unit for alcohol or per gram for tobacco. Estimates of effects were presented in 3 out of 22 sources: 2 alcohol and 1 cross-sector. For alcohol, a per unit tax was estimated to slightly reduce alcohol spending overall, with declines in all but those with the highest incomes ^36, 70^, although one grey literature paper estimated that the policy would raise tax revenue overall ^103^. A per unit tax was also estimated to lead to a reduction in alcohol-related mortality among consumers, particularly those on lower incomes ^36, 70^. For tobacco, research has begun to explore a move towards a fully specific (per gram) tax structure helping to harmonise tobacco product duty rates – at the time of the review the EU Tobacco Tax Directive required that duty rates are comprised by a combination of ad valorem (percentage of retail price) and specific (per gram) taxes.

***Multi-rate tax structures*.** Twenty-two sources: Multi-rate taxation structures apply different tax rates dependent on product type or strengths. Examples include scaled volumetric taxation ^72, 75, 76^, strength-related tax tiers ^72, 75, 95, 112^ and a duty band for heated tobacco products ^86, 89, 115^. Taxes might also be structured to maximise revenue return ^51^. Looking across alcohol and tobacco, one paper suggested restructuring fiscal policies for unhealthy commodities by coordinating tax and pricing policy across food, soft drinks, alcohol and tobacco ^94^. Estimates of effects were presented in 6 out of 22 sources: 6 alcohol. For alcohol, the >7.5% Alcohol By Volume (ABV; a measure of alcoholic strength) and <2.8% ABV beer tax bands implemented in the UK in 2011 were predicted to have only a small effect on affordability due to the relatively small volume of beer sold in these duty brackets and due to the premium prices of high strength craft beers ^104, 105^. Similarly, the 2019 6.9–7.5%ABV cider tax band set at £50.71 per hectolitre was predicted to increase the price of a 3 litre bottle of cider by only 31p (or 9%) ^76^. UK multi-rate tax structures for beer and cider were observed to have reduced the market share of high-strength products and increased that of low-strength products ^72^. Further, modelling has shown that taxing strong spirits at a relatively high rate is an effective way to reduce alcohol volume purchased by heavy drinkers without imposing large costs on lighter drinkers ^95^.

***Supplementary tax*.** Four sources: A supplementary, or added, tax could be introduced in addition to excise duty. Estimates of effects were presented in 3 out of 4 sources: 2 alcohol, 1 cross-sector. For alcohol, a 4% ad valorem alcohol sales tax (or retail excise duty) on product value added after duty at time of purchase (i.e., applied at the same time as VAT). The 4% was predicted to prompt small changes in expenditure with little subgroup variation ^36^. A 2p per unit ring-fenced treatment tax on off-trade sales was predicted to add 4p to a pint, 18p to a bottle of wine and 56p to a standard bottle of spirits, raising c. £155m p.a. 2015–17, £290m p.a. 2018–20; £410m p.a. 2021–23; £520m p.a. 2024 onwards ^116^. While a 2.5% consequential impact tax on the purchase price of ‘lifestyle self-abuse’ goods was predicted to raise £0.45bn on tobacco and £2.32bn on alcohol and a related 15% tax on advertising and sponsor spend was predicted to raise £120m ^117^.

***International harmonisation*.** Four sources: International harmonisation of tax rates and/or structure between nation states was examined in 4 sources. For alcohol, options were the introduction of minimum European tax levels on alcoholic beverages ^49^ and joining the World Wine Trade Group to harmonise standards with other wine-exporting countries and lower trade costs ^96^. For tobacco, tax rates could be harmonised across borders, to ensure FM and HRT equivalence and implement minimum excise levels as with the UK’s MET ^40, 86^. There was also a proposal to include raw tobacco in the Tobacco Tax Directive as an excisable product ^86^. No sources reported on observed or estimated effects of harmonisation.

### Industry measures

Three main categories of industry measures were identified, with these being discussed more frequently for tobacco than for alcohol.

***Industry levies*.** Ten sources: Industry levies were discussed for tobacco and for alcohol and tobacco together. A levy is a cost levied on manufacturers or retailers of alcohol or tobacco products as a percentage of revenue or profit in addition to excise duty and VAT. For tobacco, a levy could be implemented in four ways. (1) As a surcharge on corporation tax: 28% or 33% as per banking sector, user fee, or licensing charge ^86, 87^. (2) As a profit-based levy targeted at UK market operations which might reduce industry incentives to maximise profits and invest in marketing ^118^. (3) As a revenue-based levy, entailing a fee per stick or a proportion of total sales revenue by company ^118^. (4) As a fixed tax revenue levy raising £500m total with proportion allocated on sales volume ^65, 87– 89, 119, 120^. For alcohol and tobacco, a levy could comprise a 15% tax on industry spend on advertising and sponsorship, reducing industry incentives to produce and promote products and estimated to generate £194m per annum ^117^. Or it could be introduced in the form of a public health supplement as trialled in Scotland where a 13% levy was imposed on large retailers (rateable value >£300,000) selling both alcohol and tobacco products ^122^.

***Wholesale price cap accompanied with a rise in excise duty*.** Six sources: A small group of papers introduced the novel idea of a price cap on tobacco products. This option would involve a limit placed on the wholesale price at which manufacturers can sell their products to retailers. This measure would have to be combined with an equivalent excise duty rise to prevent the retail price from falling ^90, 91, 101, 102, 118, 121^. The aim of this measure would be to reduce manufacturer profits and increase the price of the cheapest products through the rise in excise duty. It has been estimated that a system of price-cap regulation in the UK would raise around £500 million per year ^121^.

***Subsidies.*** Seven sources: For alcohol only, the literature examined the role of industry or sector subsidies implemented via the excise duty system to encourage or discourage particular businesses or products. An example of this is the existing Small Breweries Relief, where initial production of 0–5000hl attracts a 50% duty cut, with a sliding scale up to 60,000hl ^104, 123, 124^. For beer, the small brewery subsidy introduced in the UK in 2002 was found to increase short- (but not long-) term profits and may have increased entry into the market, but did not have an effect on survivorship ^123, 124^. A similar duty relief for small cider producers could also be introduced ^72^. A more novel idea is to introduce tax incentives for the on-trade, for example via a differential beer duty or lower rate of VAT for draft beer, which has been found to be likely to have a negligible or small impact on tax receipts but to impact consumption at an individual level among male drinkers aged 35+ from lower socio-economic groups ^46^.

### Hypothecation motives for changing tax

Twenty-one sources: As with industry measures, hypothecation interventions were discussed more frequently for tobacco than for alcohol. Hypothecation would see revenue from excise duties, industry levies, or other tax ‘hypothecated’– i.e., reserved – for a particular purpose (e.g., for spending on alcohol or tobacco treatment services or for the National Health Service (NHS)). For alcohol, options explored in the literature included the additional revenue from tax increases being hypothecated for support for families affected by alcohol use, particularly those on low incomes or to offset NHS costs of alcohol-related harm ^38, 52, 53^. A more specific proposal was for the introduction of a ring-fenced treatment tax on every unit of alcohol sold off-trade to fund effective abstinence-based rehabilitation centres ^116^. For tobacco, similarly, tobacco tax revenues could be hypothecated for the NHS or for treatment and cessation services ^45, 62, 66, 67, 83, 86, 92^. Allocating tobacco duty revenue to the NHS has been shown to be likely to have positive health effects, generating additional quality-adjusted life years ^83^. Revenue from industry levies could be hypothecated in the same way ^61, 65, 70, 86– 89, 118– 120^. Finally, revenue from a cross-cutting consequential impact tax or Public Health Supplement would increase overall revenue which could be hypothecated for a specific purpose. For example, it is argued that the revenue generated by a consequential impact tax (£2.5bn p.a.) would enable the NHS to tackle costs of consumption, with potential greatest effect on lower socio-economic groups ^117^. When implemented, Scotland’s Public Health Supplement raised £95.9m over three years, but the hypothecation element of the policy was ultimately dropped ^122^.

### Policy objectives

Eighty-four sources referred to one or more objective for changing tax on alcohol or tobacco, of which 29 were peer reviewed journal articles. Objectives were grouped thematically into five categories ( Table 2). First, changing product affordability, including price and relative price (23 sources). Second, changing consumer behaviour, including changing consumption and supporting consumers to quit or change their consumption (47 sources). Third, changing health outcomes, including reducing and preventing harm and reducing health inequalities (45 sources). Fourth, changing economic outcomes, such as raising revenue and reducing financial costs to society (40 sources). Fifth, changing industry behaviour (26 sources). This category included objectives relating to restricting industry – reducing illicit trade, industry profits and industry manipulation of pricing to reduce the intended effects of tax policy. It also included objectives which were more sympathetic to industry including supporting industry and encouraging product reformulation.

**Table 2.** Policy objectives of tax options differentiated by product type.

		Policy options			
Objective category	Policy objectives	Excise duties	Tax structures	Industry measures	Hypothecation interventions
Change affordability	Change product affordability				
Change consumer behaviour	Change consumption				
	Support consumers				
Change health outcomes	Reduce/prevent harm				
	Reduce health inequalities				
Change economic outcomes	Raise revenue				
	Reduce financial costs to society				
Change industry behaviour	Reduce illicit trade				
	Reduce industry profits				
	Tackle industry manipulation of tax policy				
	Support industry				
	Encourage product reformulation				

**Table:** KEY.

Alcohol	Tobacco	Alcohol & Tobacco	Neither

In terms of the relationship between the types of tax policy options and specific objectives, all four options were perceived as aiming to change economic outcomes and support consumers for both alcohol and tobacco ( Table 2). Excise duties and structural tax reforms were perceived to change affordability and consumption, again for both substances. In relation to industry measures, objectives concerning reducing illicit trade and tackling industry pricing strategies were only mentioned in tobacco sources while objectives concerning incentivising product reformulation and supporting industry were only mentioned in alcohol sources.

### Policy mediators

Policy mediators were identified in 81 sources (52 grey, 29 peer reviewed). We grouped these thematically into four categories: politics and society (35 sources); policy mix (36 sources); consumers (24 sources); industry (24 sources) (see *Extended data* ^35^ and described below). Much of the commentary on mediators was general. Where it is specific to alcohol or tobacco, or to a particular type of intervention, this is indicated in the text.

***Politics and society*.** Thirty-five sources: Alcohol and tobacco tax policy debates and decisions were reported to be influenced by economic arguments ^44, 73, 78, 106^, perceptions of public acceptability and historical precedent ^49, 50, 54, 67, 71, 72, 92, 94, 112, 121^, and by narratives regarding potential regressive effects ^117^. Public, medical and political support for tax interventions ^50, 70, 72, 87, 107, 108, 112, 117, 119, 122^ and government buy-in to evidence on health benefits of tax measures ^113, 114, 116^ were regarded as important for policy action. For alcohol, political narratives mediate against tax increases: social importance of the pub trade ^79, 104^, political and economic importance of the industry ^55, 78, 104^, need to protect the ‘moderate’ or ‘responsible’ drinker ^19, 52, 104, 105^, and the idea that tax is a barrier to investment, reduces sales and costs jobs ^48, 52^. Industry influence can perpetuate these narratives and tax policy outcomes via partnerships with government (alcohol) or non-industry bodies (tobacco), lobbying, campaigns, arguments and legal action ^17, 39, 52, 61, 74, 76, 79, 101, 113, 114, 118^. Brown identified industry power as “one of the biggest barriers” to tackling the affordability of alcohol ^17^. For tobacco, political leadership and public opinion were considered to have supported sustained tax rises in the UK since the 1990s and may lead to the introduction of new measures such as levies ^39, 92^. Overall, more alcohol than tobacco sources considered politics and society to be a barrier to tax interventions (9 tobacco, 21 alcohol, 5 mixed).

***Policy mix*.** Thirty-six sources: Synchronising alcohol and tobacco tax changes with investment in prevention, education, enforcement of minimum age policies and treatment might increase effectiveness in reducing consumption and associated harms ^39, 44, 45, 62, 67, 68, 71, 84, 99, 112, 119^. Investing in enforcement efforts and sanctions (e.g. Trading Standards Agency) can discourage illicit trade ^40, 43, 48, 61, 63, 71, 84, 90, 99^. At the structural level, EU regulations have restricted government freedom to change alcohol and tobacco tax rates and structures or to introduce levies ^46, 56, 70, 83, 101, 103– 106, 109, 118, 124^. However, ‘Brexit’ (Britain’s exit from the European Union) may facilitate a review of these restrictions and could lead to higher prices on imported alcohol products ^55, 70, 72, 96^.

***Consumers*.** Twenty-four sources: Alcohol and tobacco tax effectiveness is dependent on consumer responses ^37, 41– 43, 57, 63, 67, 68, 71, 78, 97, 105, 110^. For example, demand is affected by income, prosperity, poverty, age, gender and level of drinking ^19, 37, 41, 47, 63, 85^. Demand is also affected by the wider economic context (e.g. by recession) ^50, 69, 77^. Consumers may mediate excise duty changes by changing the products they consume (e.g. cheaper but not necessarily weaker alcohol products, higher tar or nicotine cigarettes, cheaper brands or forms of tobacco such as HRT, bulk buying in carton packs) ^19, 43, 63, 68, 90, 95, 98, 105^. Consumers may also change their purchasing practices (e.g. supermarkets not convenience stores, duty-free, social network or illicit sources, where enforcement does not limit availability) ^68, 71, 90, 93^. Finally, consumers may change how they consume, for example smoking less cigarettes more intensively ^63, 68^.

***Industry*.** Twenty-four sources: Industry mediate tax policy post-implementation via tax pass-through and pricing strategies ^43, 56, 58, 61, 63, 80, 86, 98, 101, 102, 110, 113^. Modifying the tax pass-through to product sales prices can take two forms: “under-shifting” in which industry decreases the profits that they make from product sales in order to mitigate the effects of tax increases on product sales prices; “over-shifting” in which industry increases the profits that they make from product sales at the same time as a tax increase, which could act to mitigate any lost profits from decreases in consumer demand (see Wilson *et al.* ^125, 126^ for recent analyses of tax pass-through for alcohol and tobacco in the UK). For example, producers (and retailers for alcohol) under-shift tax onto cheaper products and over-shift tax onto premium products, widening the gap between them ^47, 53, 64, 71, 79– 81, 83, 90, 100, 111^. Supermarkets shift alcohol tax rises onto non-alcohol products (i.e. loss-leading) ^53, 56^. Multi-national companies can offset lost profits in the UK in other markets ^102^. Companies can reduce their duty liability by modifying their timetable for tax clearance (e.g. releasing more product for sale before a duty escalator increase) ^75, 100^. Concern was expressed that small incremental tax increases can drive industry profits as they support the industry’s pricing strategy ^91^. For alcohol, attempts to support profitability of small and medium sized businesses via the tax system are unlikely to be successful when supply chains are dominated by more powerful businesses ^123^. Industry also mediate tax via product reformulation and marketing ^72, 112^. For tobacco, industry over-supply of low-demand markets and poor supply chain control can facilitate availability of illicit products in the marketplace ^83^.

## Discussion

### Summary of findings

The literature on alcohol and tobacco tax policy in the UK focuses mainly on common measures such as duty rate changes and duty escalators. However, other tax policy options, such as structural reforms, industry measures and hypothecation, are also explored. Some, such as fully ‘specific’ or equivalent tax structures – e.g., per unit or per gram – are discussed across alcohol and tobacco. Others, such as multi-rate structural reforms and industry subsidies relate mainly to alcohol, while industry levies, price caps and minimum excise taxes are mainly discussed in relation to tobacco. The explanation for this difference is likely to lie in the different configuration of objectives and mediating factors for alcohol versus tobacco tax policy in the UK. Each of them share changing affordability, improving health, reducing health inequalities and raising revenue as objectives. However, in terms of consumption, alcohol interventions are focused on reducing harmful consumption, while tobacco control aims to reduce the prevalence of smoking (i.e., encourage quitting, reduce uptake). Tax policy narratives identified as influencing policy decisions reflect this difference. Dominant alcohol narratives described in the literature focus on harmful versus moderate drinkers and relative acceptance of the role of the alcohol industry. Those for tobacco are characterised by universal acceptance of the harms of smoking and exclusion of the tobacco industry from policy debates.

Thus, a rich seam of evidence and ideas relating to alcohol and tobacco tax policy options exists for the UK. These are summarised in *Extended data* ^35^. and have been used to directly inform the SYNTAX project: firstly, informing the development of the qualitative interview topic guide and briefing for interview participants ^127^; second, in modelling alcohol and tobacco tax options in the later part of the SYNTAX project. In terms of gaps in the evidence base, the relatively small volume of peer-reviewed literature on industry measures and hypothecation mechanisms is worthy of note. Further, while structural reform is a common theme for discussion, the associated literature presents mainly modelling-based research, rather than real-world evidence, and reports reflect uncertainty generated by Britain’s exit from the EU ('Brexit'). Following Brexit, the UK Government has introduced structural changes to the duty structure for alcohol products, creating a standardised series of tax bands based on alcohol by volume and introducing tax relief for small producers and on products sold in the on-trade venues, such as pubs ^28^. These changes to the alcohol tax system provide an opportunity to collect real-world evidence on its impacts. For tobacco tax, Brexit gives the UK more freedom to change the level and structure of tax on tobacco products, e.g. the UK could move to a fully specific system of taxation, and Brexit could also allow the UK to directly regulate tobacco pricing by the use of policies such as minimum unit pricing ^128^.

Few sources discussed alcohol and tobacco tax-related policies in the same space, except in general policy terms, and no research has yet attempted to model the interactions between alcohol and tobacco production, consumption and tax policy in the way proposed in our previous work ^7^. This previous work reported the findings of a workshop on the joint tobacco–alcohol policy system with UK academics and policy professionals. The findings illustrated the need to model the effects of policies that target tobacco and/or alcohol consumption in common terms because this allows fair comparisons between the effects of changes to tobacco policy and alcohol policy. Furthermore, to understand the effects of policy changes on socio-economic or health inequalities, it is important to understand how changes to tobacco and alcohol policy might affect individuals differently, thinking particularly of the characteristics of people who both smoke and drink to harmful levels. The tobacco and alcohol fields tend to operate in substance specific research which, while acknowledging the links between these two behaviours and their determinants, rarely looks at the two issues together. This is particularly important given the comorbidity and multiplicative risks of alcohol and tobacco consumption, particularly in relation to cancer ^129, 130^. It is also important give evidence of similarities between the practices and tactics of the alcohol and tobacco industries ^131^.

From this review of the literature, there is clear demand for conceptualising and understanding tax policy within its wider context as ‘tax and spend’, rather than solely as a means of raising revenue. There is also a debate to be had about whether this is then interpreted as a Pigouvian tax system which aims to meet the costs of externalities of product use and/or as a hypothecated public health tax system which aims to invest in prevention and treatment. In order to take this debate forward, there is more work to be done to understand the more complex landscape of policy options described in this review and how they may interact with and fit with each other. At present, it is very unclear from the literature what ‘effective’ combinations of policy options might look like, and these are likely to vary depending on the policy objective(s) being prioritised.

### International relevance

Tax has been shown to be among the most effective ways to change consumption of harmful commodities ^132– 134^. For those readers interested in a global overview of alcohol and tobacco tax options, it is worth looking at Chaloupka *et al.*, ^135^. The paper covers a similar spread of policy options and concludes that excise taxes are a ‘powerful tool’ for reducing tobacco use and excessive drinking and that their potential to ‘significantly reduce consumption and save lives remains high’. The scope of our review was purposefully restricted to papers which focused wholly or partly on the UK. This was to ensure that the review’s findings were both relevant to the UK’s policy context and manageable within the timeframe available to deliver the review. To address this limitation, the SYNTAX project’s International Advisory Panel were invited to comment on the findings. They flagged a number of relevant complementary examples from around the world that we outline below in relation to the four policy themes identified in this review:

First, on tobacco excise duty rates, combining a high tax floor, an escalator and occasional surprise large tax increases is thought to be an effective way to reduce consumption ^20^. Tax increases are expected to have a progressive rather than regressive impact on health because tobacco consumption is higher among people with lower socio-economic status who also have greater price sensitivity ^135^.

Second, on tax structures, evidence from Sweden has shown that a shift to a tax strategy based on alcohol strength led to an increase in floor prices and a substantial reduction in consumption of the cheaper segment of the market ^136, 137^. In addition, there is evidence from Australia that the reach of differential tax rate for beers is highly dependent on the thresholds set for each tax tier ^138^. 

Third, on industry measures, taxing inputs, such as materials or labour, might also reduce industry profitability. For alcohol and tobacco, our review identified options such as levies that take money from the industries to help reduce the societal costs of their products. However, due to the perceived social and economic importance of alcohol in contrast to tobacco, policy options also discussed supporting pubs and small breweries. More work is needed to understand the influence of the price elasticity of demand for alcohol and tobacco products on the potential for industry measures to raise revenue for government given that the industry might pass the costs onto consumers. It is also unclear how tax changes on alcohol and tobacco products could affect the wider economy via impacts on the labour market and productivity across multiple sectors.

Fourth, on hypothecation, New Zealand, the USA and Canada all offer examples of hypothecation of a fixed tax on alcohol. New Zealand funded the Alcohol Advisory Council (ALAC); Washington State has funded alcohol-related research; and Québec funds Edu Alcool, a not-for-profit education and prevention organisation. There is also research on the relative acceptability of hypothecated versus across-the-board taxes showing that the former is more popular ^139^. In terms of equity, using revenues to fund programmes that benefit the poor increases their progressive impact, as in the Philippines’ universal healthcare programme which is funded by tobacco taxes ^135^. In terms of effectiveness, the case of tobacco shows that the market share of the illicit trade tends to be lower in jurisdictions with higher tax rates, particularly where tax administration and enforcement is effective ^135^. This indicates that the effectiveness of tax policy could be enhanced by hypothecating tax revenue for the enforcement of tax regulations.

### Strengths and limitations

The strengths of the study lie, first, in the rapid but systematic approach which has been rigorously followed ^140^. This approach allowed the review to capture the extent and nature of the work that had been produced on alcohol and tobacco tax policy options relevant to the UK in 2018, which we used as part of the progression of research in the SYNTAX project. The review findings were used to prepare to speak to alcohol and tobacco tax experts about the options for changing tax on alcohol and tobacco, which we did in 2018. Thus, whilst the 2018 end date of the search is a limitation of the study because it means that the study does not capture evidence published from 2018 to the date of publishing in 2023, as the review formed part of the larger SYNTAX project, the rapid scoping review methodology instrumentally enabled progression of the research. It facilitated the project team’s understanding of the literature and allowed us to consider tax options, not only in relation to their broad characteristics, but also with reference to technical detail, objectives, evidence of effects and mediators.

The SYNTAX research project’s focus on tax policies meant that non-tax price measures such as minimum unit pricing fell outside the scope of the review, even though tax and non-tax price measures are often intrinsically linked in policy discussions. In terms of limitations of the methodology, the review did not critically appraise the literature and embraced a variety of sources that may have been excluded from a more traditional systematic review, e.g. policy briefings and model-based predictions. However, this decision is in line with recommended best practice for rapid scoping reviews ^31, 32^. It is mitigated somewhat in this case by the research team’s recording and reporting of types of sources, research funding and methods used. In doing so, the review reflects the nature of both peer-reviewed evidence and the wider policy debate on alcohol and tobacco taxation discussed in the grey literature. It also responds to the need identified by Parkhurst and Abeysinghe to think about evidence quality in terms of appropriateness as well as rigor in relation to evidence-based policy as opposed to evidence-based medicine ^141^. 

## Conclusions

This review has clearly identified a contemporary set of policy objectives, interventions and related mediating factors that were summarised in a briefing to alcohol and tobacco tax policy stakeholders ahead of interviews for the SYNTAX project about the options for changing tax on alcohol and tobacco ^127^. The differences between alcohol and tobacco tax interventions and debates suggest an opportunity for cross-policy learning. There is currently little literature/evidence which considers joint effects of tax options for alcohol and tobacco, despite evidence that co-consumption multiplies risks to health. Modelling the impact of these alcohol and tobacco policy options to better understand their relative impact would provide information to help decide among the available options.

## Data Availability

Figshare: SYNTAX Rapid Scoping Review: PRISMA Scoping Review checklist and Supplementary Information https://doi.org/10.15131/shef.data.22032644 ^35^ This project contains the following underlying data: Supplementary Information - SYNTAX Rapid Scoping Review.pdf PRISMA checklist - SYNTAX Rapid Scoping Review.pdf Figshare: PRISMA checklist for ‘Options for modifying UK alcohol and tobacco tax: A rapid scoping review of the evidence over the period 1997–2018’. https://doi.org/10.15131/shef.data.22032644 ^35^. Data are available under the terms of the Creative Commons Attribution 4.0 International license (CC-BY 4.0).
