# Supplementary material for: Options for modifying UK alcohol and tobacco tax: A rapid scoping review of the evidence over the period 1997–2018
Source: NIHR Open Res. 2023 Oct 30;3:26. Originally published 2023 May 16. [Version 3] doi: 10.3310/nihropenres.13379.3 (PMC10593339; doi:10.3310/nihropenres.13379.3)
Supplement: Peer review history

# Reviewer report: Reviewer response for version 3

**DOI:** 10.3310/nihropenres.14660.r30659

**Published:** 2023-11-16

**Authors:** Lana Elliott

**Affiliations:** 1 Queensland University of Technology, Brisbane, Queensland, Australia

The authors should be congratulated on this robust paper and their dedication to the peer review process. I have no further suggestions for improvements.

Are the rationale for, and objectives of, the Systematic Review clearly stated?

Yes

Is the statistical analysis and its interpretation appropriate?

Not applicable

Are sufficient details of the methods and analysis provided to allow replication by others?

Yes

Are the conclusions drawn adequately supported by the results presented in the review?

Yes

Reviewer Expertise:

Health taxes, commercial determinants of health & political economy analysis

I confirm that I have read this submission and believe that I have an appropriate level of expertise to confirm that it is of an acceptable scientific standard.

# Reviewer report: Reviewer response for version 2

**DOI:** 10.3310/nihropenres.14638.r30487

**Published:** 2023-10-18

**Authors:** G. Emmanuel Guindon

**Affiliations:** 1 McMaster University, Hamilton, Ontario, Canada

The authors have adequately addressed all my initial comments. I have two additional minor comments:

 “ Fourth, changing economic outcomes, such as raising revenue and reducing financial costs to society, also known as externalities.”

- First, economic outcomes and financial outcomes are not necessarily the same. What is being described are financial outcomes, not really economic outcomes. Similarly, there are different types of externalities, financial externalities being only one type (other types include paternalistic (caring) externalities and option-value externalities). Second, raising revenue has little to do with externalities.

“Tax increases are expected to have a progressive rather than regressive impact because tobacco consumption is higher among people with lower socio-economic status who also have greater price sensitivity.”

-  It’s worth clarifying what is meant by “regressive/progressive.” Regressive/progressive in

health?  Regressive/progressive in income? I believe clearly progressive in health but not necessarily progressive in income.

 Minor formatting issue:

 Politics and society, policy mix, consumers, and industry are subsections of “Policy mediators”

Are the rationale for, and objectives of, the Systematic Review clearly stated?

Yes

Is the statistical analysis and its interpretation appropriate?

Not applicable

Are sufficient details of the methods and analysis provided to allow replication by others?

Yes

Are the conclusions drawn adequately supported by the results presented in the review?

Partly

Reviewer Expertise:

Health economics; economics of health behaviours

I confirm that I have read this submission and believe that I have an appropriate level of expertise to confirm that it is of an acceptable scientific standard.

# Response

**Published:** 2023-10-23

**Authors:** Duncan Gillespie

**Affiliations:** University of Sheffield, UK

Thank you for the additional minor comments, which we have now addressed.

# Reviewer report: Reviewer response for version 2

**DOI:** 10.3310/nihropenres.14638.r30486

**Published:** 2023-10-10

**Authors:** Lana Elliott

**Affiliations:** 1 Queensland University of Technology, Brisbane, Queensland, Australia

The authors have addressed concerns raised in my initial review and should be congratulated on their insightful paper.

Are the rationale for, and objectives of, the Systematic Review clearly stated?

Yes

Is the statistical analysis and its interpretation appropriate?

Not applicable

Are sufficient details of the methods and analysis provided to allow replication by others?

Yes

Are the conclusions drawn adequately supported by the results presented in the review?

Yes

Reviewer Expertise:

Health taxes, commercial determinants of health & political economy analysis

I confirm that I have read this submission and believe that I have an appropriate level of expertise to confirm that it is of an acceptable scientific standard.

# Reviewer report: Reviewer response for version 1

**DOI:** 10.3310/nihropenres.14510.r29497

**Published:** 2023-09-14

**Authors:** G. Emmanuel Guindon

**Affiliations:** 1 McMaster University, Hamilton, Ontario, Canada

This is a well written study on an important topic.

**Modelling studies.**

 Modelling studies assume that prices decrease demand, and as such, do not provide any evidence that prices affect demand. Throughout the article, modelling studies seem to be conflated with studies that quantitatively examined the relationship between prices and demand using real-world data. This article would greatly benefit from a discussion and clear distinction between the two types of studies.  As it stands, many statements are simply not supported by the evidence presented.

 Similarly, causal language is used throughout and at times, not supported by the evidence presented.

 Examples include:

- P3 “ *This is supported by evidence that increases in product prices decrease demand9–14, especially in socio-economically disadvantaged groups15–17*.”
- P6 “ *Evidence of policy effects was presented in 20 out of 37 sources: 8 alcohol, 11 tobacco and 1 cross-sector (shown in bold in Table 1).*”
- Table 1

**Modelling the interactions between alcohol and tobacco production, consumption and tax policy.**

- Recent evidence does not suggest meaningful cross-price elasticities between alcohol and tobacco. See for example: https://doi.org/10.1016/S0140-6736(16)32420-5 ^1^ and https://doi.org/10.1111/add.15966 ^2^

**Non-tax price measures**

- The omission of non-tax price measures such as minimum pricing should be discussed as a limitation as they are often intrinsically linked in policy discussions.

**Brexit:**

- A short discussion of the Brexit implication for alcohol and tobacco taxation would be helpful.

**Minor comments/edits:**

- PICO framework:
  - Consider briefly describing the PICO framework the first time it is mentioned.
- P4: [Only applies to Member States apply excise duty < EUR 115].
  - A word seems to be missing.
- Acronyms:
  - Spell out acronyms the first time it appears (e.g., NHS)

Are the rationale for, and objectives of, the Systematic Review clearly stated?

Yes

Is the statistical analysis and its interpretation appropriate?

Not applicable

Are sufficient details of the methods and analysis provided to allow replication by others?

Yes

Are the conclusions drawn adequately supported by the results presented in the review?

Partly

Reviewer Expertise:

Health economics; economics of health behaviours

I confirm that I have read this submission and believe that I have an appropriate level of expertise to confirm that it is of an acceptable scientific standard, however I have significant reservations, as outlined above.

## References

[1] : A rapid evidence review of the effectiveness and cost-effectiveness of alcohol control policies: an English perspective. *Lancet* .2017;389(10078) : 10.1016/S0140-6736(16)32420-5 1558-1580 10.1016/S0140-6736(16)32420-5 27919442

[2] : Prices, taxes and alcohol use: a systematic umbrella review. *Addiction* .2022;117(12) : 10.1111/add.15966 3004-3023 10.1111/add.15966 35661298 PMC9796894

# Response

**Published:** 2023-09-18

**Authors:** Duncan Gillespie

**Affiliations:** University of Sheffield, UK

Dear Dr Guindon,

 Many thanks for your review of our article “Options for modifying UK alcohol and tobacco tax: A rapid scoping review of the evidence over the period 1997–2018”. We found the comments very helpful and tried hard to act on each of them fully. Below we describe our responses (in bold) to your comments (in italics).

 Yours sincerely,

 Duncan Gillespie

Comments from the reviewer:

*This is a well written study on an important topic.*

*Modelling studies.*

*Modelling studies assume that prices decrease demand, and as such, do not provide any evidence that prices affect demand. Throughout the article, modelling studies seem to be conflated with studies that quantitatively examined the relationship between prices and demand using real-world data. This article would greatly benefit from a discussion and clear distinction between the two types of studies.  As it stands, many statements are simply not supported by the evidence presented.*

**Thank you for highlighting that more clarification is needed here. The distinction between studies that we focus on in our presentation of results is whether or not a study presented any quantitative policy effect sizes vs. whether it was a purely qualitative paper/report. It is important to acknowledge that modelling studies are often based on a range of empirical evidence, including price elasticities of demand estimated using real world data. In the manuscript text we have clarified that due to the scoping nature of our study, we simply distinguish studies that are qualitative from studies that present policy effect sizes, which could be effect sizes observed from statistical analyses or effect sizes predicted by computer simulation models.**

*Similarly, causal language is used throughout and at times, not supported by the evidence presented.*

**The paper has now been reviewed for this causal language and edited, see below for edits to the examples mentioned.**

*Examples include:*

*P3 “This is supported by evidence that increases in product prices decrease demand9–14, especially in socio-economically disadvantaged groups15–17.”*

**This is now edited to: “In general, there is evidence that increases in product prices decrease demand,9-14 especially in socio-economically disadvantaged groups.15-17”**

*P6 “Evidence of policy effects was presented in 20 out of 37 sources: 8 alcohol, 11 tobacco and 1 cross-sector (shown in bold in Table 1).”*

**This is now edited to: “Observed or predicted estimates of policy effects were presented in 20 out of 37 sources: 8 alcohol, 11 tobacco and 1 cross-sector (shown in bold in Table 1).”**

**

*Table 1*

*Modelling the interactions between alcohol and tobacco production, consumption and tax policy.*

*Recent evidence does not suggest meaningful cross-price elasticities between alcohol and tobacco. See for example:* *https://doi.org/10.1016/S0140-6736(16)32420-5* *[1] and* *https://doi.org/10.1111/add.15966* *[2]*

**Thank you for pointing to these two evidence reviews.  We were aware of both and have now included them as references.  We would not conclude that there is “no suggestion of meaningful cross-price elasticities between alcohol and tobacco”.  Rather we conclude that there is an absence of good studies on the topic.  The two evidence reviews are basically pointing to a lack of robust studies.**

**This is exactly why, as part of the SYNTAX project, we have produced new estimates of own- and cross-price effects for 10 alcohol products and 2 tobacco products for the UK, leading to a new 12x12 price elasticity matrix (currently under review).**

*Non-tax price measures*

*The omission of non-tax price measures such as minimum pricing should be discussed as a limitation as they are often intrinsically linked in policy discussions.*

**Thank you for highlighting this – text has now been added to the limitations in the Discussion.**

*Brexit:*

*A short discussion of the Brexit implication for alcohol and tobacco taxation would be helpful.*

**Thank you for highlighting this – text has now been added to the Discussion that gives a brief update on the situation for tobacco and alcohol policy.**

*Minor comments/edits:*

*PICO framework:*

*Consider briefly describing the PICO framework the first time it is mentioned.*

**Thank you for this suggestion - The following sentence has now been added to the “Data charting” section of the methods. “The PICO framework is a commonly used model for structuring the key elements of studies in terms of the Population, Intervention, Comparison, Outcome(s).”**

*P4: [Only applies to Member States apply excise duty < EUR 115].*

*A word seems to be missing.*

**This is now edited in Box 1 to “Only applies to Member States that apply excise duty < EUR 115].”**

*Acronyms:*

*Spell out acronyms the first time it appears (e.g., NHS)*

**The paper has now been reviewed for acronyms to ensure that they are spelled out the first time they appear.**

*References*

*1. Burton R, Henn C, Lavoie D, O'Connor R, et al.: A rapid evidence review of the effectiveness and cost-effectiveness of alcohol control policies: an English perspective.Lancet. 2017; 389 (10078): 1558-1580 PubMed Abstract | Publisher Full Text*

*2. Guindon GE, Zhao K, Fatima T, Garasia S, et al.: Prices, taxes and alcohol use: a systematic umbrella review.Addiction. 2022; 117 (12): 3004-3023 PubMed Abstract | Publisher Full Text*

# Reviewer report: Reviewer response for version 1

**DOI:** 10.3310/nihropenres.14510.r29494

**Published:** 2023-06-27

**Authors:** Lana Elliott

**Affiliations:** 1 Queensland University of Technology, Brisbane, Queensland, Australia

Thank you for the opportunity to review this incredibly interesting research paper. The paper is well written, methodically sound and the authors should be commended on their comprehensive analysis. I have outlined below some overarching and section-by-section suggestions for strengthening this body of work:

**Overarching comments:**

- Given Brexit and substantial changes in the health taxes space between 2018 and 2023, I wonder if updating this search would be benefit. If not, I think it’s important for the authors to outline what specific lessons still hold from 2018 and what may have shifted given changes over the last 5 years. This could be done in a similar manner to which the ‘international relevance’ subsection has been written.

**Abstract:**

- The paper’s abstract is clear and well-constructed.

**Keywords:**

- I would suggest including ‘commercial determinants of health’ as an additional key word

**Plain language summary:**

- Under “Why we did it”, I would suggest adjusting the sentence that begins ‘Increasing alcohol tax…’ to ‘Increasing alcohol and tobacco taxes has been shown to reduce their consumption.’

**Introduction:**

- P1: ‘Policy system’ feels a little bit odd. I would suggest the authors amend this sentence to read ‘Of the available interventions to jointly address alcohol and tobacco related harms, increasing is considered among the most effective.’
- P2: ‘after manufacture’ should read ‘after manufacturing’
- A brief clause explaining why the duty escalator on alcohol was removed would be beneficial to understanding dynamics in this paragraph.
- Please include the VAT rate in the final sentence of P2.

**Methods:**

- Review approach: It’s a little unclear as to whether you’re summarising a modified Tricco approach in the sentence beginning ‘This involves’. Adjusting it to state ‘This involved…’ would clarify that it is the process your paper followed if this is the intention of the sentence.
- My understanding of scoping reviews is that quality appraisal isn’t typical so I’m a little unclear as to why it’s been outlined as a specific modification. 

**Results:**

- An in-text breakdown of how many studies were predictive/modelling vs. those based on observed/empirical evidence would be helpful.
- ‘Change duty rates on specific products’ subheading P3: HRT and FM have already been outlined in full in the previous subsection so acronyms alone would suffice
- ‘Multi-rate tax structures’ subheading: Can the authors please provide an explanation of the acronym ABV
- Table 2: This table is really helpful in understanding the alignment between policy options, objectives and categories. Switching the colour of the ‘alcohol and tobacco’ category however would help slightly with readability.
- ‘Policy mix’ subsection: Can the authors provide an update on whether there has been any review of these measures in light of Brexit?
- ‘Industry’ subheading: an more in-depth explanation of what is meant by under- and over-shifting tax may be helpful to readers with less familiarity of this area.

**Discussion:**

- Some detail on the forthcoming structural reforms to alcohol duties in the UK would be beneficial to this section to understand the types of data that would be considered valuable and how they may be fed into future changes in this space.
- The observation that little research has considered alcohol and tobacco in the same space is interesting but could use some further unpacking. The authors have highlighted why this may be a good idea but it would also be worth including a couple of sentences of perhaps why this hasn’t been done to date.

**Strengths and limitations:**

- I would have expected to see the 2018 end date of the search included as a potential limitation given the changes that have occurred globally, nationally and in the health taxes space between now and then.

**International relevance:**

- I would suggest shuffling this subsection to before the study’s strengths and weaknesses so its relevance can be reflected on as a strength/weakness.
- It may be worth summarising Chaloupka et al.’s work more concisely and seeing if it can be integrated into the paragraph above a little more. They’re really interesting arguments to summarise but, at the moment, it almost reads as if this paper is finishing with a summary of another paper. Reflecting on each point in relation to what this paper adds (or doesn’t) in relation to these arguments may be another way to go about it.

Are the rationale for, and objectives of, the Systematic Review clearly stated?

Yes

Is the statistical analysis and its interpretation appropriate?

Not applicable

Are sufficient details of the methods and analysis provided to allow replication by others?

Yes

Are the conclusions drawn adequately supported by the results presented in the review?

Yes

Reviewer Expertise:

Health taxes, commercial determinants of health & political economy analysis

I confirm that I have read this submission and believe that I have an appropriate level of expertise to confirm that it is of an acceptable scientific standard, however I have significant reservations, as outlined above.

# Response

**Published:** 2023-09-18

**Authors:** Duncan Gillespie

**Affiliations:** University of Sheffield, UK

Dear Ms Elliot,

 Many thanks for your review of our article “Options for modifying UK alcohol and tobacco tax: A rapid scoping review of the evidence over the period 1997–2018”. We found the comments very helpful and tried hard to act on each of them fully. Below we describe our responses (in bold) to your comments (in italics).

 Yours sincerely,

 Duncan Gillespie

Comments from the reviewer:

*Overarching comments:*

*Given Brexit and substantial changes in the health taxes space between 2018 and 2023, I wonder if updating this search would be benefit. If not, I think it’s important for the authors to outline what specific lessons still hold from 2018 and what may have shifted given changes over the last 5 years. This could be done in a similar manner to which the ‘international relevance’ subsection has been written.*

**Thank you for giving us the opportunity to improve the paper in this regard. As this paper is published as a record of research undertaken in the SYNTAX project, on which subsequent parts of that project were built, we will not be updating the search undertaken in 2018. We have improved the explanation of this in the Introduction and Discussion.**

**In addition, we agree that it is important to outline relevant recent changes to the context for tobacco and alcohol tax policy.**

**For alcohol in the UK, there has been a major tax policy change in the intervening period - the reform of duty structure for alcohol products. This has created a standardised series of tax bands based on alcohol by volume and has introduced tax relief for small producers and on products sold in the on-trade venues, such as pubs. This policy change is highlighted in the Introduction and we have now also added text to the Discussion.**

**For tobacco, there is an active policy debate about changes to tobacco taxation in the UK, but no major changes have yet been introduced. We have added text and a new reference to the Discussion that outlines the policy debate.**

*Keywords:*

*I would suggest including ‘commercial determinants of health’ as an additional key word*

**Thank you – a note to the editors has been made to change the keyword of “commercial influences on health” to “commercial determinants of health”.**

*Plain language summary:*

*Under “Why we did it”, I would suggest adjusting the sentence that begins ‘Increasing alcohol tax…’ to ‘Increasing alcohol and tobacco taxes has been shown to reduce their consumption.’*

**This change has now been made in the manuscript and the online submission system.**

*Introduction:*

*P1: ‘Policy system’ feels a little bit odd. I would suggest the authors amend this sentence to read ‘Of the available interventions to jointly address alcohol and tobacco related harms, increasing is considered among the most effective.’*

**This change has now been made.**

*P2: ‘after manufacture’ should read ‘after manufacturing’*

**This change has now been made.**

*A brief clause explaining why the duty escalator on alcohol was removed would be beneficial to understanding dynamics in this paragraph.*

**Thank you for pointing out the need for clarification here – we have added this clause and a new reference. The removal of the duty escalator on alcohol came about due to a political decision by the UK Government. The reference added gives further explanation of the context and model-based estimates of the health and economic impacts of the decision to remove the duty escalator.**

*Please include the VAT rate in the final sentence of P2.*

**The VAT rate of 20% has been added.**

*Methods:*

*Review approach: It’s a little unclear as to whether you’re summarising a modified Tricco approach in the sentence beginning ‘This involves’. Adjusting it to state ‘This involved…’ would clarify that it is the process your paper followed if this is the intention of the sentence.*

**“Involves” has now been changed to “involved” to clarify that this is the process that our paper followed.**

*My understanding of scoping reviews is that quality appraisal isn’t typical so I’m a little unclear as to why it’s been outlined as a specific modification.*

**Thank you for highlighting the need for clarification on the review approach. We have now edited the Review approach section and added a new reference. The methodology used for scoping studies is highly variable – the new reference explains this “Arksey H, O'Malley L. Scoping studies: towards a methodological framework. International journal of social research methodology. 2005;8(1):19-32.” Our study used a modified rapid review approach drawn from two papers cited in the article by Tricco et al. The Tricco et al papers show that most of the rapid review methods did conduct quality appraisal. However, due to the scoping nature of our review that had the objective of identifying policy options for tobacco and/or alcohol tax, quality appraisal was not needed. The exclusion of quality appraisal is therefore outlined as a specific modification to the Tricco et al rapid review approach.**

*Results:*

*An in-text breakdown of how many studies were predictive/modelling vs. those based on observed/empirical evidence would be helpful.*

**Thank you for suggesting this – however, we feel this is a difficult distinction to make in relation to the broad objectives of our scoping review study and because models are often based on a range of empirical evidence. The distinction between studies that we focus on in our presentation of results is whether or not a study presented any quantitative policy effect sizes vs. whether it was a purely qualitative paper/report. We have clarified this in the manuscript text at the first mention of Table 1 in which a breakdown of sources is presented.**

*‘Change duty rates on specific products’ subheading P3: HRT and FM have already been outlined in full in the previous subsection so acronyms alone would suffice*

**This change has now been made.**

*‘Multi-rate tax structures’ subheading: Can the authors please provide an explanation of the acronym ABV*

**This has now been clarified by adding “Alcohol By Volume (ABV; a measure of alcoholic strength)” at the first mention of the acronym ABV.**

*Table 2: This table is really helpful in understanding the alignment between policy options, objectives and categories. Switching the colour of the ‘alcohol and tobacco’ category however would help slightly with readability.*

**Thank you for highlighting this as an issue – we have made a note to the editors to change the colour scheme.**

*‘Policy mix’ subsection: Can the authors provide an update on whether there has been any review of these measures in light of Brexit?*

**Please see our response to your overarching comment above.**

*‘Industry’ subheading: an more in-depth explanation of what is meant by under- and over-shifting tax may be helpful to readers with less familiarity of this area.*

**Thank you for highlighting this – a brief explanation and further references have been added to this section.**

*Discussion:*

*Some detail on the forthcoming structural reforms to alcohol duties in the UK would be beneficial to this section to understand the types of data that would be considered valuable and how they may be fed into future changes in this space.*

**We agree that this is interesting, however there is not the space in this paper to give a proper treatment of this issue. Our research team has developed a large-scale project to evaluate the impacts of the structural reforms to alcohol duties, which covers a wide range of interconnected data sources.**

*The observation that little research has considered alcohol and tobacco in the same space is interesting but could use some further unpacking. The authors have highlighted why this may be a good idea but it would also be worth including a couple of sentences of perhaps why this hasn’t been done to date.*

**Thank you for suggesting we expand on this. We have added text that explain the findings of a workshop that we held with UK academics and policy professionals to understand why alcohol and tobacco research has not often been considered in the same space and why doing so might be desirable.**

*Strengths and limitations:*

*I would have expected to see the 2018 end date of the search included as a potential limitation given the changes that have occurred globally, nationally and in the health taxes space between now and then.*

**The strengths and limitations section has now been edited to clarify this issue and to explain why the study is limited in having a 2018 end date of the search. Also see our response to your overarching comment above.**

*International relevance:*

*I would suggest shuffling this subsection to before the study’s strengths and weaknesses so its relevance can be reflected on as a strength/weakness.*

**This section has now been moved to above the strengths and limitations section.**

*It may be worth summarising Chaloupka et al.’s work more concisely and seeing if it can be integrated into the paragraph above a little more. They’re really interesting arguments to summarise but, at the moment, it almost reads as if this paper is finishing with a summary of another paper. Reflecting on each point in relation to what this paper adds (or doesn’t) in relation to these arguments may be another way to go about it*

**This text is now referred to more concisely and integrated with the paragraph on international relevance. The new paragraph structures the arguments in relation to the four main policy options identified by our review.**
